# Distinct Expression Pattern of Epigenetic Machinery Genes in Blood Leucocytes and Brain Cortex of Depressive Patients

**DOI:** 10.1007/s12035-018-1406-0

**Published:** 2018-10-30

**Authors:** Romain Rey, Jean-Christophe Chauvet-Gelinier, Marie-Françoise Suaud-Chagny, Sylviane Ragot, Bernard Bonin, Thierry d’Amato, Jean-Raymond Teyssier

**Affiliations:** 1 INSERM, U1028; CNRS, UMR5292; Lyon Neuroscience Research Center, Psychiatric Disorders: from Resistance to Response Team, F-69000 Lyon, France; 2 University Lyon 1, F-69000 Villeurbanne, France; 3 Schizophrenia Expert Centre, Le Vinatier Hospital, Bron, France; 4 INSERM U1028; CNRS UMR5292; Université Claude Bernard Lyon 1; Centre de Recherche en Neurosciences de Lyon, Equipe PSYR2; Centre Hospitalier Le Vinatier, Pole Est, Centre Expert Schizophrénie, 95 boulevard Pinel BP 30039, 69678 Bron Cedex, France; 5 Psychiatry Unit, Neurosciences Department, Le Bocage University Hospital, Marion Building, Dijon, France; 6 Laboratory of Psychopathology and Medical Psychology (IFR 100), Bourgogne University, Dijon, France; 7 Department of Genetics and Laboratory of Molecular Genetics, University Hospital, Dijon, France

**Keywords:** Depression, Epigenesis, Genetic, Brain, Leucocytes, Gene expression

## Abstract

In major depressive disorder (MDD), altered gene expression in brain cortex and blood leucocytes may be due to aberrant expression of epigenetic machinery coding genes. Here, we explore the expression of these genes both at the central and peripheral levels. Using real-time quantitative PCR technique, we first measured expression levels of genes encoding DNA and histone modifying enzymes in the dorsolateral prefrontal cortex (DLPFC) and cingulate cortex (CC) of MDD patients (*n* = 24) and healthy controls (*n* = 12). For each brain structure, transcripts levels were compared between subject groups. In an exploratory analysis, we then compared the candidate gene expressions between a subgroup of MDD patients with psychotic characteristics (*n* = 13) and the group of healthy subjects (*n* = 12). Finally, we compared transcript levels of the candidate genes in blood leucocytes between separate samples of MDD patients (*n* = 17) and healthy controls (*n* = 16). In brain and blood leucocytes of MDD patients, we identified an overexpression of genes encoding enzymes which transfer repressive transcriptional marks: HDAC4-5-6-8 and DNMT3B in the DLPFC, HDAC2 in the CC and blood leucocytes. In the DLPFC of patients with psychotic characteristics, two genes (KAT2A and UBE2A) were additionally overexpressed suggesting a shift to a more transcriptionally permissive conformation of chromatin. Aberrant activation of epigenetic repressive systems may be involved in MDD pathogenesis both in brain tissue and blood leucocytes.

**Electronic supplementary material:**

The online version of this article (10.1007/s12035-018-1406-0) contains supplementary material, which is available to authorized users.

## Introduction

Major depressive disorder (MDD) affects more than 350 million people worldwide and is one of the most common mental illnesses [1]. It is now considered as the third leading cause of DALYS (disability-adjusted life years) worldwide and will become the second leading cause of disability by 2030 [2]. MDD is also responsible for a dramatic increase in mortality due to suicide, but also to somatic diseases (especially metabolic and cardiovascular pathologies) [3]. In this respect, MDD is now considered as a systemic disease, in which pathological processes take place not only at the cerebral level but also in peripheral tissues [4, 5]. Despite its high prevalence and major medical impact, the understanding of MDD pathogenesis is still limited. Increasing knowledge of its basic features represents thus a major issue, especially because of the suboptimal efficiency of the current treatments.

The quantification of candidate gene expression and transcriptional profiling using RNA extracted from postmortem brain tissues and blood leucocytes of MDD patients have characterized differential expression of genes involved in neurogenesis, synaptogenesis, neuroplasticity, cell signaling, cell survival, neuro-endocrine functionality, and inflammation [6–15]. Evidence from rodent models and correlative observation in humans has recently suggested that epigenetic dysregulations may contribute to the genetic changes observed in MDD patients [16, 17]. Since epigenetic modifications dynamically integrate environmental influences at genome level [18, 19] and exert long-lasting effect on gene expression, dysregulation of the epigenetic enzymes which regulate the transcriptional state of the chromatin is likely to be involved in MDD pathogenesis, at both the brain and peripheral level.

The epigenetic mechanisms regulate gene expression by covalently adding and removing various chemical groups at multiple sites of both the chromatin proteins and the DNA molecule. These modifications produce complex and dynamic signalization patterns, which are recognized by the gene expression complex as repressive or permissive marks. The most specific mark of expressed genes is histone acetylation which exerts a permissive action by opening the conformation of the chromatin and is catalyzed by Histone Acetylases (including KAT2A). Although less studied, ubiquitination of histone by UBE2A is also a tag of transcriptional activation. On the other hand, the permissive state is reversed by multiple Histone Deacetylases (HDAC2,4,5,6,8). Methylation of histone by Histone Methylases (including EHMT2) is also recognized as a specific tag of epigenetic repression. At gene level, DNA methylation (catalyzed by DNA methyltransferases) mediates transcriptional repression [20].

Aberrant DNA methylation and histone acetylation caused by altered expression of DNA methyltransferases (DNMTs) and histone deacetylases (HDACs), as well as other histone post-translational modifications such as acetylation, methylation, and ubiquitination have been associated with MDD pathogenesis [16, 21, 22]. However, information about the role of the epigenetic machinery in the pathophysiology of MDD is still scarce and mostly derived from experimental results in rodents [23–25]. So far, only four human studies have quantified the mRNA levels of genes encoding DNMTs or HDACs in blood leucocytes by the real-time quantitative PCR technique (qPCR). In these studies, depressive state was characterized by a downregulation of HDACs (including SIRT1, 2, and 6) and DNMT1, and an upregulation of HDAC2, HDAC5, and DNMT3B [26–29]. To date, only two studies have explored the expression of genes coding for HDACs in postmortem brain tissue of depressed patients [30, 31]. Using brain samples obtained from the Dallas Brain Collection, Covington et al. reported an increased expression of HDAC2 in the nucleus accumbens of MDD patients compared to control subjects [30]. Moreover, in postmortem dorsolateral prefrontal cortex (DLPFC) samples collected at the Human Brain collection Core (HBCC) and at the Brain and Tissue Bank for Developmental Disorders, Schroeder et al. recently observed an upregulation of HDAC2 in MDD patients vs. control subjects [31]. To assess the clinical and biological significance of these findings, they should be replicated and extended. Moreover, in order to evaluate the neurobiological relevance of the changes identified in blood cells, they should be compared to those observed in other brain regions relevant to MDD, especially the frontocingulate cortex, known to play a crucial role in the cognitive and affective symptoms [32, 33]. This dual brain-blood approach is of importance to assess the functional genetic changes which underlay the systemic mechanisms of MDD.

Using real-time quantitative PCR, we aimed to identify altered expression of genes encoding epigenetic enzymes both at the central and peripheral level in depressive patients. The candidate genes code for enzymes involved in the establishment of the following epigenetic marks: maintenance (DNMT1) and de novo (DNMT3A, DNMT3B) DNA methylation; histone deacetylation (HDAC2, HDAC4, HDAC5, HDAC6, HDAC8); histone acetylation (KAT2A); histone methylation (EHMT2) and histone ubiquitination (UBE2A). Although KAT2A, EHMT2, and UBE2A are crucially involved in the regulation of gene transcription, to our knowledge, their expression pattern in MDD has not been previously evaluated. The main hypothesis of this project was that the expression of genes encoding epigenetic machinery enzymes was altered in MDD patients compared to healthy control subjects. First, we quantified the expression of the candidate genes in the DLPFC and cingulate cortex (CC) of MDD patients (*n* = 24) and unaffected control subjects (*n* = 12). Second, to look for similarities between the gene expression patterns in brain and blood, we quantified the expression of the same genes in blood leucocytes of a separate sample of MDD patients (*n* = 17) and matched healthy control subjects (*n* = 16). Finally, as an exploratory hypothesis restricted to the brain tissue samples, we postulated that the expression of the candidate genes was specifically altered in a subgroup of MDD patients with psychotic characteristics (*n* = 13) compared to healthy control subjects (*n* = 12). For this project, we obtained postmortem brain samples (from the Stanley Medical Research Institute brain collection) and blood leucocytes (from the University Hospital of Dijon-France) allowing us to perform original analyses in order to extend current knowledge on epigenetic machinery coding genes and replicate previous findings in independent samples.

## Material and Methods

### Brain Samples

The brain RNAs extracted from postmortem dorsolateral prefrontal cortex (DLPFC-BA 46) and the whole CC have been donated by the Stanley Medical Research Institute brain collection. The depression collection consisted of 36 subjects: 13 MDD patients without psychosis, 11 MDD patients with psychotic characteristics, and 12 unaffected control subjects. These samples were collected, with informed consent from next-of-kin, by participating medical examiners between January 1995 and June 2002. The samples were all collected, processed, and stored in a standardized way. Exclusion criteria for all subjects were as follows: significant structural brain pathology on postmortem examination by a qualified neuropathologist or by premortem imaging, history of significant focal neurological signs premortem, history of a central nervous system disease that could be expected to alter gene expression in a persistent way, documented IQ < 70, poor RNA quality. Additional exclusion criteria for unaffected controls were age less than 30, substance abuse within 1 year of death, or significant alcohol-related changes in the liver. Diagnoses were made by two senior psychiatrists, using DSM-IV criteria and based on medical records and, when necessary, telephone interviews with family members. Diagnoses of unaffected controls were based on structured interviews by a senior psychiatrist with family member(s) to rule out Axis I diagnoses. To our knowledge, the brain samples analyzed in the present study never resulted in published data regarding epigenetic machinery coding genes.

### Blood Leucocytes

The leucocyte RNAs have been extracted after venipuncture from the buffy coat of 33 peripheral blood samples obtained from 17 patients with MDD and 16 matched unaffected controls. Seventeen female MDD patients were recruited upon admission in the Psychiatry Unit (University Hospital of Dijon) for a depressive episode diagnosed by a psychiatrist with the Structured Clinical Interview for DSM-IV-TR (SCID) and the Mini-International Neuropsychiatric Interview (MINI). The severity of the symptoms was assessed with the Hamilton depression and anxiety rating scales (HAM-D and HAM-A, respectively). The life-long duration of the disease and the number of episodes were evaluated from clinical interviews and medical records. Subjects with any comorbid psychiatric disorder diagnosed by the MINI were excluded, except for anxiety symptoms. Medical examination, interview, medical records, and laboratory tests were used to exclude somatic pathology, especially cardiovascular and metabolic diseases. Sixteen healthy control women recruited among hospital staff members were matched for age and alcohol consumption. Controls were healthy active women without somatic disease, psychiatric diagnostic, medical treatment, or significant medical history. All the included participants were of Caucasian origin. Patients and controls provided a written informed consent to participate in this study, and the detailed protocol was approved by the relevant ethical review board (CPP of the University Hospital of Dijon).

### RNA Isolation from Human Circulating Leucocytes and RNA Quality Control

A total of 12 mL of venous peripheral blood was collected from fasting patients and healthy controls at 8 am, in two 6 mL EDTA tubes. The RNA isolation procedure was performed within 1 h after blood collection. For each sample, the buffy coat containing leucocytes was collected by centrifugation after lysis of red blood cells. Total RNA was extracted from leucocytes using Trizol reagent (Life Technologies, USA) according to the manufacturer’s protocol. DNase treatment was performed for each sample.

The RNA concentration was determined by measuring the optical density at 260 nm wavelengths using a NanoDrop 8000 spectrophotometer (Thermo Scientific, USA). Purity and quality were assessed by absorbance at UV_260/280_; a ratio between 1.8 and 2.0 was needed for further analysis. The quality of RNA, computed as RIN (RNA Integrity Number), was assessed with an Agilent 2100 Bioanalyser (Agilent Technologies, USA) using RNA 6000 Nano Chips (Agilent Technologies, USA). The mean RIN values for blood leucocytes varied from 9 to 9.8 (mean ± SD, 9.38 ± 0.26), those for the DLPFC from 8.1 to 9.5 (mean ± SD, 8.45 ± 0.38) and those for the CC from 7.9 to 9.1 (mean ± SD, 8.32 ± 0.56).

### mRNA Determination

Each sample of total RNA was reversed transcribed using the QuantiTect Reverse Transcription Kit (Qiagen S.A., France) according to the manufacturer’s protocol. The cDNA solution was stored at − 20 °C. The level of transcripts in the brain tissue and blood leucocytes was measured by the real-time quantitative polymerase chain reaction (rQ-PCR) method exactly as previously described [34]. The qPCR reaction was run on a Light Cycler 480 II (Roche Diagnostics, France) using the QuantiTect SYBR Green PCR Kit (Qiagen S.A., France) according to the manufacturer’s instructions. The cycling conditions were the following: 95 °C for 15 min (activation of HotStart Taq DNA polymerase), followed by 45 cycles of PCR amplification process including denaturing at 94 °C for 15 s, annealing at 60 °C for 1 min, and extension at 72 °C for 40 s. A final step (95 °C for 30 s, 50 °C for 1 min, and gradual heating to 95 °C) was set up to generate melting curves which checked the specificity of the amplification products. Forward and reverse primers were designed for each target mRNA using Primer3 software (Whitehead Institute for Biomedical Research) under stringent primer picking conditions (no 3′ self-complementarity and less than 4 self-complementarities). All the PCR primer pairs used in this experiment generated amplicons between 80 and 150 base pairs. The sequence and location of the primers used for gene amplification are available upon request. The GAPDH gene has been selected as the reference (normalizing) gene using geNorm [35] in qbase+ software, version 3.1 (Biogazelle, Zwijnaarde, Belgium—www.qbaseplus.com). Implementation of geNorm in qbase+ enables the selection of the optimal, most stable reference gene from a series of tested candidate reference genes. For the DLPFC and CC tissues, we tested three commonly employed candidate reference genes (GAPDH, B2M, and HGPRT) and geNorm identified GAPDH as the most stable reference gene (geNorm M-value < 0.5). For the blood leucocytes, we tested four genes previously identified as suitable candidate reference genes in peripheral blood (DECR1, TRAP1, RPS19, and GAPDH) [36] and GAPDH was identified as the most stable reference gene (geNorm M-value < 0.5). Each qPCR plate included a “no reverse transcriptase” and no “template” control to monitor non-specific amplification, each sample was essayed in triplicate and the results were expressed as the mean of the three Ct values. The PCR efficiency has been established between 0.97 and 0.98 by the standard curve method. In the brain tissues and blood leucocytes, none of Ct values of target and reference genes was over 31 cycles of amplification.

### Statistical Analysis

XLSTAT software (XLSTAT 2017: Data Analysis and Statistical Solution for Microsoft Excel. Addinsoft, Paris, France, 2017) was used to perform statistical analysis. Sociodemographic and clinical characteristics of subjects in the control and MDD patient groups were compared using unpaired *t* test (for age, brain pH, and postmortem interval) and Fisher exact test (for gender and presence of drug, alcohol, and tobacco consumption). ANCOVA testing was conducted to evaluate whether each candidate gene expression level was impacted by disease-relevant covariates. In the DLPFC, the CC, and blood samples, ANCOVA analysis was conducted using the mRNA level of each candidate gene as a dependent variable and with age, disease duration, number of depressive episodes, and scores at the Hamilton scales as quantitative independent variables, and sex, suicide status, presence of a lifetime antipsychotic treatment, presence of tobacco, alcohol and drug consumption as categorical independent variables.

For the quantification of the transcripts, the Ct values of target and reference genes in the MDD and control groups have been analyzed and compared by the Relative Expression Software Tool (REST 2008 V2. 0.7) based on the mathematical model provided by Pfaffl et al. which is considered as a standard for analyzing relative gene expression [37]. This mathematical model is based on the PCR efficiencies and the mean crossing point deviation between two groups. REST software allows for a normalization of the target genes with a reference gene. Subsequently, REST software uses a Pair Wise Fixed Reallocation Randomization Test to determine whether a significant difference exists between groups while taking issues of reaction efficiency and reference gene normalization into account. Randomization test with a pair-wise reallocation is the most appropriate approach for relative expression ratio determination. Such a test makes no assumption about the distribution of observations which would always be questionable for gene expression measurements [37]. Through this mathematical model, REST provides a robust data analysis and accurate *p* levels. For the main and exploratory analyses, gene expression comparisons were considered to be statistically significant for *p* < 0.05.

## Results

### Demographic and Clinical Characteristics

The demographic and clinical characteristics of the subjects who provided brain tissue are summarized in Table 1. For MDD patients, the age of disease onset ranged from 13 to 59 years (mean ± SD, 29.9 ± 11.9) and the duration of illness varied from 0.1 to 31 years (mean ± SD, 12.3 ± 7.6). All patients were treated by antidepressants whose dosage was not available. No significant difference in demographic and clinical characteristics (age, sex, race, brain pH, postmortem interval, presence of alcohol consumption, presence of drug consumption) was found between the control and MDD patient groups. For the exploratory analysis, when we compared control subjects and MDD patients with psychosis, there was no significant difference in demographic and clinical characteristics (Table S1). ANCOVA testing of each potential covariate (age, sex, presence of alcohol and drug consumption in all included subjects (i.e., MDD patients and controls); disease duration, suicide status, presence of a lifetime antipsychotic treatment in the MDD patients only) revealed no significant impact on transcript levels of candidate genes in the DLPFC, nor in the CC.

**Table 1:** Characteristics of the subjects who provided brain tissue

	Controls	All MDD	*p* value*
	(*n* = 12)	(*n* = 24)	
Age (mean years ± SD)	46.8 ± 12.1	42.2 ± 10.9	0.20
Sex (M/F)	8/4	13/11	0.72
Caucasian Race	11	23	1
Suicide	–	17	–
Age of onset (mean years ± SD)	–	29.9 ± 11.9	–
Disease duration (mean years ± SD)	–	12.3 ± 7.6	–
Alcohol use (%)	58.3	66.6	0.72
Drug use (%)	33	33	1
Brain pH (mean ± SD)	6.64 ± 0.18	6.65 ± 0.15	0.82
PMI (mean hours ± SD)	25.3 ± 10.6	29.7 ± 12.4	0.30

*PMI* postmortem interval

*Unpaired *t* tests and Fisher exact tests were conducted to assess group differences for continuous and discrete variables, respectively

The demographic and clinical characteristics associated with the blood samples are summarized in Table 2. The diagnostic of MMD was established for the first time in 12 patients (first episode) and five patients had experienced more than three episodes. The duration of the current depressive episode was less than 6 months for all the patients. In the MDD group, the HAM-D scores varied from 17 to 29 (mean ± SD, 25.3 ± 4.3) and the HAM-A scores from 15 to 37 (mean ± SD, 24.4 ± 5). Twelve patients received treatment by antidepressant and anxiolytic drugs, three patients were included before treatment initiation and the therapeutic status was unknown for two patients. Presence of antipsychotic treatment was not available. No participants reported current use of drug of abuse. No significant difference in demographic and clinical characteristics (age, presence of tobacco consumption, presence of drug consumption) was found between the control and MDD patient groups. ANCOVA testing of each potential covariate (age, presence of alcohol and tobacco consumption in all included subjects (i.e., MDD patients and controls); number of depressive episodes, disease duration, scores at the Hamilton scales, suicide status in the MDD patients only) revealed no significant impact on transcript levels of candidate genes in the blood leucocytes.

**Table 2:** Characteristics of the subjects who provided the blood samples

	Controls	MDD	*p* value*
	(*n* = 16)	(*n* = 17)	
Age (mean years ± SD)	37.6 ± 5.2	39.5 ± 9.9	0.33
Disease duration (mean years ± SD)	–	7.9 ± 11.3	–
Depressive episodes (mean number ± SD)	–	2 ± 1.9	–
Suicide attempts (%)	–	53	–
HAM-D score (mean total score ± SD)	–	25.1 ± 3.8	–
HAM-A score (mean total score ± SD)	–	24.9 ± 4.9	–
Alcohol use (%)	43.8	41.2	0.52
Drug use (%)	0	0	1
Tobacco use (%)	0	23.5	0.10

*HAM-D* and *HAM-A* Hamilton depression and anxiety rating scales, respectively

*Unpaired *t* tests and Fisher exact tests were conducted to assess group differences for continuous and discrete variables, respectively

### Dorsolateral Prefrontal Cortex

**Fig. 1:**
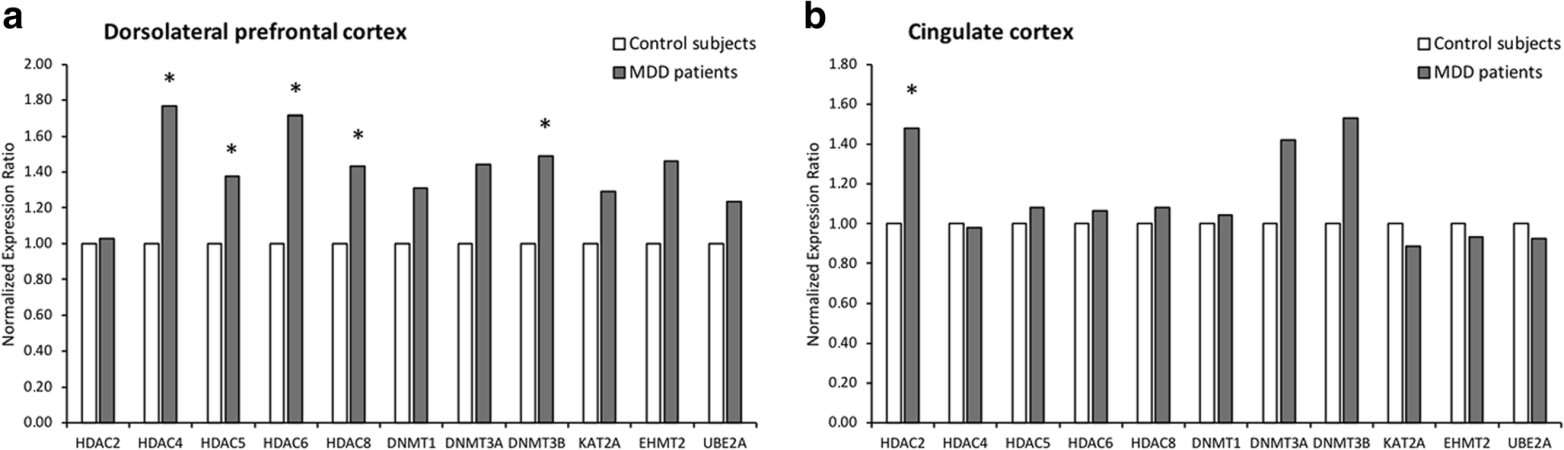
Normalized expression ratio (NER) of the candidate genes in the brain tissues of control subjects and MDD patients. For each candidate gene **a** in the dorsolateral prefrontal cortex and **b** in the cingulate cortex, NER was obtained by REST software based on the PCR efficiencies and the 2^−(ΔΔCt)^ method, with GAPDH as the reference normalizing gene. *p < 0.05 vs controls

Compared to controls, MDD patients exhibited a set of genes significantly upregulated in the DLPFC: HDAC4, HDAC5, HDAC6, HDAC8, and DNMT3B (by 77, 38, 72, 43, and 49%, respectively; *p* < 0.05) (Fig. 1a). The detailed results are summarized in Table S2.

The same set of genes was found significantly upregulated in the DLPFC of the subgroup of MDD patients with psychotic characteristics compared to control subjects: HDAC4, HDAC5, HDAC6, HDAC8, and DNMT3B (by 83, 46, 83, 66, and 40%, respectively; *p* < 0.05). Furthermore, in MDD patients with psychosis, the mRNA level of UBE2A and KAT2A was increased (by 53 and 43%, respectively; *p* < 0.05) compared to controls (Table S3).

### Cingulate Cortex

Compared to controls, expression of HDAC2 was upregulated in the CC of MDD patients (48%; *p* < 0.05) (Fig. 1b). The detailed results are summarized in Table S2.

In the subgroup of MDD patients with psychotic characteristics compared to control subjects, there was a significant upregulation of HDAC2 expression (53%; *p* < 0.05) and DNMT3A (60%; *p* < 0.05) (Table S1).

### Blood Leucocytes

**Fig. 2:**
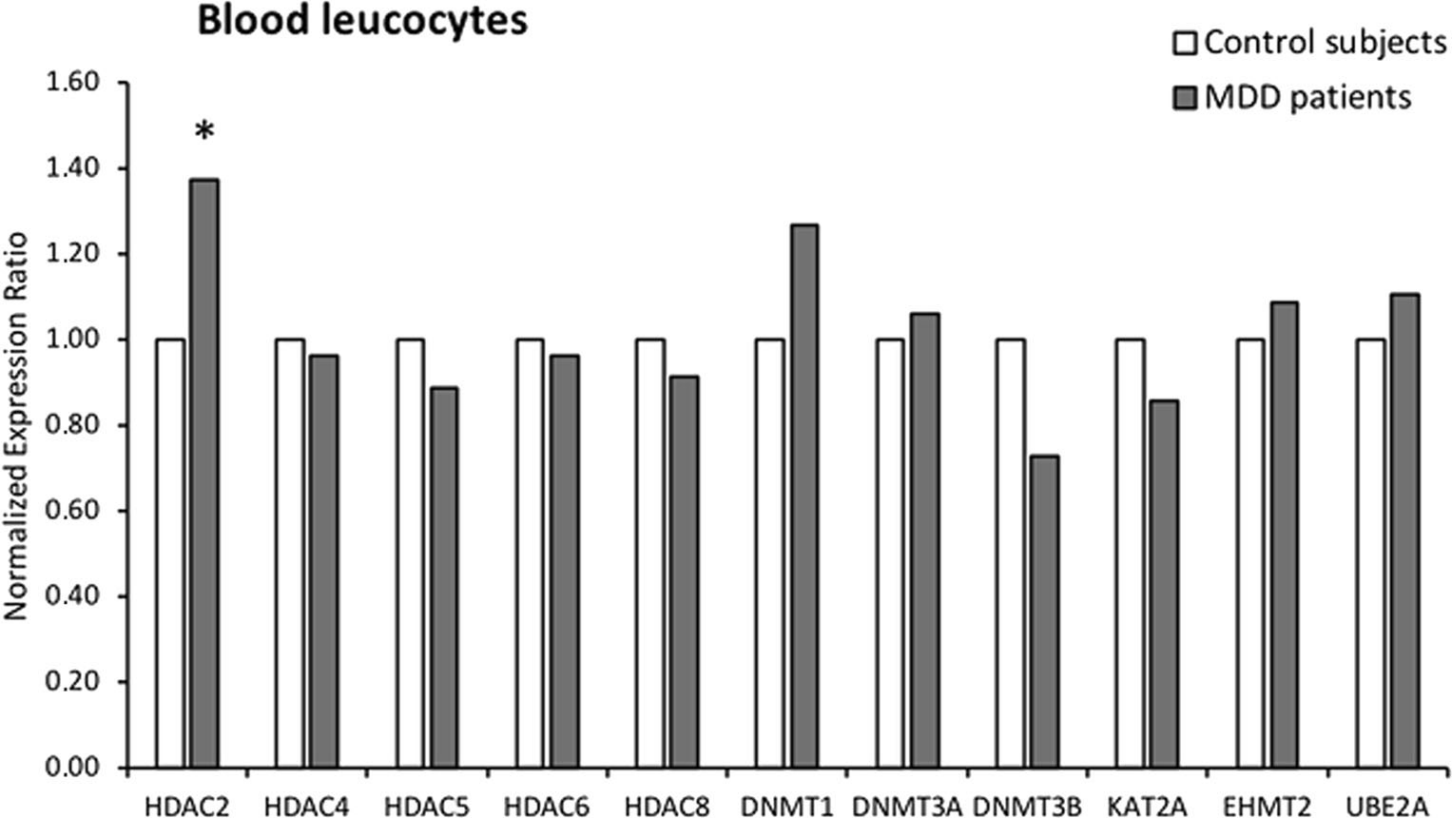
Normalized expression ratio (NER) of the candidate genes in the blood leucocytes of control subjects and MDD patients. For each candidate gene in the blood leucocytes, NER was obtained by REST software based on the PCR efficiencies and the 2^−(ΔΔCt)^ method, with GAPDH as the reference normalizing gene. *p < 0.05 vs controls

The only significant change observed in the leucocytes of patients with MDD was an upregulation by 37% (*p* < 0.05) of the HDAC2 gene expression (Table S4) (Fig. 2).

## Discussion

In both brain and peripheral blood cells of MDD patients, we report for the first time a predominant pattern characterized by overexpression of the genes encoding enzymes which transfer repressive transcriptional marks to the chromatin. In MDD patients, HDAC4-5-6-8 and DNMT3B were upregulated in the DLPFC, and HDAC2 was upregulated in the CC. At the periphery, HDAC2 was also overexpressed in blood cells of MDD patients. Our exploratory analysis in the subgroup of MDD patients with psychotic characteristics suggests that three additional genes were selectively upregulated in the brain tissue of this specific subgroup of MDD patients: KAT2A and UBE2A in the DLPFC, and DNMT3A in the CC.

In the DLPFC of MDD patients, we report for the first time the concurrent increase of the steady state mRNA levels of HDAC4-5-6-8 and DNMT3B. This suggests that MDD pathophysiology is shaped by a global epigenetic repressive program in the DLPFC. This is consistent with a previous study reporting hypermethylation of the promoter region of P11 gene in the prefrontal cortex of an animal model of depression [38], and with the only available study of the global methylation pattern in human, which showed a hypermethylation trend of various genome regions in the postmortem frontal cortex of MDD patients [39]. HDAC5 overexpression was already reported in the blood leucocytes of MDD patients [27, 29]. However, HDAC4 upregulation in MDD has not been previously reported, although it was previously observed in blood leucocytes of bipolar patients only during the depressive state [27]. Moreover, upregulation of HDAC6 and HDAC8 reported in MDD patients contrasts to their downregulation in blood leucocytes of bipolar patients in depressive and remissive state [27]. Upregulation of DNMT3B and downregulation of DNMT1 were previously reported in peripheral white blood cells of current MDD patients [28] and in the frontopolar cortex of depressed suicide victims, whereas expression of both genes was reduced in the amygdala [40]. In the present study, no change was observed in the brain expression of DNMT1. This result does not support a central role for this gene in the DLPFC of MDD patients, although recent data from rodent models reported antidepressant-like properties of DNMT1 inhibition [41, 42].

HDAC4-5-6-8 are known to be critically involved in various aspects of synaptic plasticity and neurogenesis [43–46]. HDAC4 is implicated in both age- and Alzheimer disease-associated cognitive decline [47–49] and DNMT3B in cognitive ability of psychotic patients [50] and healthy subjects [51]. Hence, the overexpression of these genes may participate to the disruption of neural plasticity occurring in the DLPFC of MDD patients and thus to cognitive impairment [32]. Additional mechanisms may be involved since HDAC6 regulates the glucocorticoid receptor signaling pathway [52] and the inflammatory response in astrocytes [53]. Further studies are needed to replicate these findings and to explore the link between increased HDACs/DNMT3B expression in the DLPFC of MDD patients and cognitive dysfunction.

Another significant finding of this study was an upregulation of HDAC2 expression in the CC and blood leucocytes of MDD patients, but no significant change in the DLPFC. Our results are consistent with those from Hobara et al. who identified blood upregulation of HDAC2 and HDAC5 expression as a marker of the depressive state which normalized upon remission [27]. However, in contrast to Hobara et al., the present study does not find an upregulation of HDAC5 expression in blood cells. The discrepancy may be accounted for by the heterogeneity of the demographic and clinical characteristics of the patient samples: we studied a female population and the majority of our patients (70%) were experiencing a first episode of depression, while that studied by Hobara et al. included 50% of men whose MDD clinical status is not specified. There is no doubt that chronicity as well as the number, duration, and severity of depressive episodes may critically influence the epigenetic system. In the DLPFC of MDD patients, the absence of difference in HDAC2 expression compared with control subjects does not agree with previous results reporting a significant slight increase (+ 17%) for HDAC2 expression in the DLPFC of MDD patients (*n* = 135) vs. controls (*n* = 210) [31]. It is possible that our experiment was underpowered to replicate such a slight upregulation.

It has been previously reported that HDAC2 expression is altered in opposite directions depending on the considered brain region of MDD subjects. Increased HDAC2 protein level was observed in the hippocampus of a mouse model of depression [54], but reduced HDAC2 expression was observed in the nucleus Accumbens (NAc) of another depressive rodent model as well as in postmortem NAc samples of MDD patients [30]. Moreover, since HDAC inhibitor infusion in the NAc of mice exerts antidepressant effects, it has been hypothesized that HDAC2 downregulation in the NAc may constitute an individual’s adaptation to MDD [30]. The HDAC2 complex preferentially binds to promoters of numerous synaptic plasticity genes, is a key negative regulator of long-term synaptic plasticity and memory processes, and thus plays a crucial role in cognitive performance [55, 56]. Consistently, increased HDAC2 protein level in the hippocampus of a mouse model of depression is associated with cognitive decline [54]. Thus, our finding of HDAC2 overexpression in the CC and blood leucocytes of MDD patients could reflect both a failure in the implementation of an adaptive mechanism and a marker of cognitive deficits.

Alternatively, another hypothesis regarding HDAC2 overexpression could be mentioned. Increased HDAC2 expression is observed in the prefrontal cortex during the human aging process [57, 58] and its overexpression constitutes an early pathological event in Alzheimer’s disease (AD) [56, 57, 59]. The exact mechanisms through which HDAC2 expression is linked to neurodegenerative processes could involve HDAC2-binding to the promoters of synaptic plasticity genes [60] or activation of neuro-inflammatory processes [61–65]. In this hypothesis, overexpression of HDAC2 in the CC and blood leucocytes might not be etiologically related to MDD, but rather translates activation of the cell senescence pathways and neuro-inflammatory processes, known to occur in blood leucocytes and brain of MDD patients [34, 66].

As an exploratory analysis restricted to the brain tissue samples, we compared expression of the candidate genes between the subgroup of MDD patients with psychotic characteristics and healthy control subjects. Three genes were additionally upregulated in the DLPFC (UBE2A, KAT2A) and CC (DNMT3A) of patients with psychotic characteristics. Although results observed in MDD patients suggest that MDD pathophysiology is characterized by a global epigenetic repressive program, this configuration implies that presence of psychotic characteristics might be associated with a shift to a more transcriptionally permissive conformation of chromatin in the DLPFC. Among the 17 MDD patients from whom the blood samples were collected, only 2 had psychotic characteristics. Further studies in blood leucocytes of MDD patients with psychotic characteristics are needed to look for similarities between brain and blood expression alterations. However, our results are in line with recent data reporting differences in blood microRNA between psychotic and non-psychotic depression [67]. In addition to be involved in gene expression activation, UBE2A codes for the UBE2A protein, a central effector in the ubiquitin-proteasome system (UPS) which constitutes the principal mechanism for protein catabolism. Previous studies in brain and blood have reported alterations of the UPS pathway at both the mRNA and protein levels in MDD [68]. However, no study has previously identified deregulation of UBE2A in MDD with psychotic characteristics, which suggests a potential role of UPS in psychotic mechanisms [69–71]. KAT2A and DNMT3A have been identified as potential moderators of susceptibility to stressful events which represent major risk factors for MDD [72–74]. UBE2A, KAT2A, and DNMT3A are involved in neuronal plasticity and cognitive processing [75–80]. In patients with psychotic characteristics, their overexpression may thus increase and broaden cognitive impairment associated with MDD [81].

In this study, all the overexpressed genes in the brain and/or blood of MDD patients are involved in similar processes such as neurogenesis, synaptic or neural plasticity. Their altered expression may thus be functionally involved in the cognitive impairment associated with MDD. Interestingly, different altered expression patterns of epigenetic machinery genes have already been reported in other psychiatric disorders associated with cognitive impairment such as bipolar disorder, suicide, or schizophrenia [27–29, 31, 40]. Since differences in cognitive impairment between psychiatric disorders are quantitative rather than qualitative [82–84], it may be suggested that the expression pattern of epigenetic machinery genes may determine the severity of cognitive impairment. Further studies in psychiatric disorders are needed to explore this hypothesis.

In MDD patients, we did not identify the same pattern of overexpressed genes in the DLPFC and blood leucocytes, which suggests that the blood cell compartment is not a perfect surrogate of the brain but is affected by specific functional genetic changes [85–87]. Different factors may explain DLPFC and blood discrepancy in gene expressions. First, in this experiment, blood and brain samples were not derived from the same subjects. Second, since gene expression is not expected to be uniform across all the different cortical regions [31, 40, 88], blood and brain gene expression correlation may differ depending on the cortical region. Interestingly, HDAC2 overexpression in the CC was also observed in the blood leucocytes suggesting a higher comparability between blood and CC transcript levels compared to DLPFC. Finally, MDD pathogenesis may not be restricted to brain tissue as it is now considered as a “whole-body” disorder [89]. Altered gene expression in peripheral blood may reflect a distinct molecular response of the peripheral tissue specific to MDD pathophysiology or secondary to the MDD process [90]. As such, epigenetic machinery expression in blood leucocytes may provide a means of better understanding MDD pathogenesis and constitute a potential biomarker readily accessible and more sensible to the stage of illness than brain tissue.

Interpretation of the present results is affected by limiting factors. First, blood and brain tissues were obtained from two clinically heterogeneous and modest-sized MDD samples. The former was composed of rather young women mainly diagnosed with a first episode of MDD and without major comorbidity, whereas the latter included men and women with a long-lasting and severe disease. Therefore, the gene expression changes identified in the cerebral cortex may reflect the severity of the pathological process, while those observed in the blood leucocytes may be gender specific. However, previous studies investigating expression of epigenetic machinery coding genes in blood leucocytes of MDD patients did not find any significant correlation between sex and gene expression levels [26–29]. Second, in this study, detailed modalities of the treatment were not provided. A potential effect of antidepressant or antipsychotic treatment on gene expression could not be ruled out although previous studies did not find any significant correlation between dosages of antidepressant medications and expression levels of HDACs or DNMTs in blood leucocytes [26–29]. Moreover, recent results report no relationship between antidepressant or antipsychotic status and HDAC2 expression in the DLPFC of MDD patients [31]. Consistently, in this study, ANCOVA testing with the brain tissues data did not find a significant impact of antipsychotic status on gene expression. Third, in this study, smoking status was not available for the subjects from whom the postmortem brain samples were obtained. While ANCOVA testing did not report a significant impact of tobacco consumption on gene expression in the blood leucocytes, we could not conduct the same analysis in the DLPFC and CC. However, recent results observed no significant effect of smoking on HDACs transcript levels in the DLPFC of MDD patients [31].

## Conclusions

This study provides the first evidence that activation of distinct HDACs- and DNMTs-mediated epigenetic mechanisms in the two key regions of the frontocingulate network may contribute to MDD pathogenesis. The involved histone and DNA modifying enzymes are promising targets for future therapeutic interventions. Moreover, we observed that transcriptional regulation is altered during the MDD state both in brain tissue and blood leucocytes. Finally, we confirmed a previous observation that HDAC2 is overexpressed in the blood leucocytes of patients with current depressive episode suggesting that it may represent a clinically useful peripheral marker.

## Electronic supplementary material

ESM 1 (DOCX 13 kb)

ESM 2 (DOCX 13 kb)

ESM 3 (DOCX 13 kb)

ESM 4 (DOCX 12 kb)
