# Supplementary material for: Distinct Expression Pattern of Epigenetic Machinery Genes in Blood Leucocytes and Brain Cortex of Depressive Patients
Source: Mol Neurobiol. 2018 Oct 30;56(7):4697–707. doi: 10.1007/s12035-018-1406-0 (PMC6647377; doi:10.1007/s12035-018-1406-0)
Supplement: Supplementary file 1 — (DOCX 13 kb) [file 12035_2018_1406_MOESM1_ESM.docx]

| **Table S1** Characteristics of the control subjects and MDD patients with psychotic characteristics who provided Brain Tissue | | | |
| --- | --- | --- | --- |
|  | Controls | MDD with |  |
|  |  | psychosis | *p*-value* |
|  | (n=12) | (n=11) |  |
| Age (mean years ± SD) | 46.8 ± 10.6 | 39.6 ± 10.6 | 0.12 |
| Sex (M/F) | 8/4 | 5/6 | 0.41 |
| Caucasian Race | 11 | 10 | 1 |
| Suicide | - | 8 | - |
| Age of onset (mean years ± SD) | - | 28.6 ± 12.5 | - |
| Disease duration (mean years ± SD) | - | 11 ± 5.5 | - |
| Alcohol use (%) | 58.3 | 72.8 | 0.67 |
| Drug use (%) | 33 | 33.4 | 1 |
| Brain pH (mean ± SD) | 6.64 ± 0.18 | 6.59 ± 0.15 | 0.52 |
| PMI (mean hours ± SD) | 25.3 ± 10.6 | 33.1 ± 11.0 | 0.10 |
| *PMI* Post-Mortem Interval |  |  |  |
| * Unpaired t-tests and Fisher exact tests were conducted to assess group differences for continuous and discrete variables, respectively | | | |
