# Supplementary material for: Distinct Expression Pattern of Epigenetic Machinery Genes in Blood Leucocytes and Brain Cortex of Depressive Patients
Source: Mol Neurobiol. 2018 Oct 30;56(7):4697–707. doi: 10.1007/s12035-018-1406-0 (PMC6647377; doi:10.1007/s12035-018-1406-0)
Supplement: Supplementary file 2 — (DOCX 13 kb) [file 12035_2018_1406_MOESM2_ESM.docx]

| **Table S2** Normalized expression ratio (NER) of the candidate genes in Brain Tissue of the MDD patients relative to the control subjects | | | | | | | |
| --- | --- | --- | --- | --- | --- | --- | --- |
|  | Dorso-Lateral PreFrontal Cortex | | |  |  | Cingulate Cortex |  |
|  | MDD patients relative to control subjects | | |  | MDD patients relative to control subjects | | |
| **Gene** | **NER** | **Std. Error** | ***p*-value*** |  | **NER** | **Std. Error** | ***p*-value*** |
| HDAC2 | 1.027 | 0.665 - 1.527 | 0.836 |  | **1.48** | **0.909 - 2.591** | **0.021** |
| HDAC4 | **1.766** | **0.909 - 3.264** | **0.002** |  | 0.978 | 0.562 - 1.701 | 0.889 |
| HDAC5 | **1.378** | **0.710 - 2.390** | **0.044** |  | 1.08 | 0.821 - 1.492 | 0.383 |
| HDAC6 | **1.717** | **0.914 - 3.239** | **0.002** |  | 1.065 | 0.561 - 1.984 | 0.743 |
| HDAC8 | **1.433** | **0.759 - 2.624** | **0.039** |  | 1.083 | 0.751 - 1.598 | 0.499 |
| DNMT1 | 1.309 | 0.695 - 2.436 | 0.17 |  | 1.043 | 0.485 - 2.589 | 0.897 |
| DNMT3A | 1.442 | 0.662 - 2.915 | 0.117 |  | 1.421 | 0.698 - 2.956 | 0.08 |
| DNMT3B | **1.491** | **0.761 - 2.686** | **0.037** |  | 1.53 | 0.716 - 3.065 | 0.089 |
| KAT2A | 1.293 | 0.733 - 2.247 | 0.19 |  | 0.886 | 0.608 - 1.257 | 0.362 |
| EHMT2 | 1.459 | 0.725 - 2.948 | 0.075 |  | 0.935 | 0.458 - 1.828 | 0.748 |
| UBE2A | 1.236 | 0.590 - 2.708 | 0.401 |  | 0.926 | 0.550 - 1.446 | 0.6 |
| *NER* normalized expression ratio, *MDD* major depressive disorder | | | | | | |  |
| * Significant *p*-values set at 0.05 provided by REST software | | | | | |  |  |
