# Supplementary material for: Distinct Expression Pattern of Epigenetic Machinery Genes in Blood Leucocytes and Brain Cortex of Depressive Patients
Source: Mol Neurobiol. 2018 Oct 30;56(7):4697–707. doi: 10.1007/s12035-018-1406-0 (PMC6647377; doi:10.1007/s12035-018-1406-0)
Supplement: Supplementary file 3 — (DOCX 13 kb) [file 12035_2018_1406_MOESM3_ESM.docx]

| **Table S3** Normalized expression ratio (NER) of the candidate genes in Brain Tissue of the MDD patients with psychotic characteristics relative to the control subjects | | | | | | | |
| --- | --- | --- | --- | --- | --- | --- | --- |
|  | Dorso-Lateral PreFrontal Cortex | | |  |  | Cingulate Cortex |  |
|  | MDD patients with psychotic characteristics relative to control subjects | | |  | MDD patients with psychotic characteristics relative to control subjects | | |
| **Gene** | **NER** | **Std. Error** | ***p*-value*** |  | **NER** | **Std. Error** | ***p*-value*** |
| HDAC2 | 1.003 | 0.668 - 1.512 | 0.987 |  | **1.529** | **0.834 - 2.802** | **0.048** |
| HDAC4 | **1.83** | **1.066 - 3.125** | **0.004** |  | 0.978 | 0.562 - 1.701 | 0.889 |
| HDAC5 | **1.457** | **0.943 - 2.346** | **0.02** |  | 1.08 | 0.821 - 1.492 | 0.383 |
| HDAC6 | **1.828** | **1.089 - 3.028** | **0.003** |  | 1.065 | 0.561 - 1.984 | 0.743 |
| HDAC8 | **1.66** | **1.052 - 2.649** | **0.004** |  | 1.083 | 0.751 - 1.598 | 0.499 |
| DNMT1 | 1.232 | 0.753 - 2.123 | 0.212 |  | 1.043 | 0.485 - 2.589 | 0.897 |
| DNMT3A | 1.216 | 0.662 - 2.279 | 0.318 |  | **1.601** | **0.794 - 3.197** | **0.043** |
| DNMT3B | **1.403** | **0.820 - 2.319** | **0.038** |  | 1.53 | 0.716 - 3.065 | 0.089 |
| KAT2A | **1.429** | **0.898 - 2.246** | **0.027** |  | 0.886 | 0.608 - 1.257 | 0.362 |
| EHMT2 | 1.373 | 0.801 - 2.299 | 0.084 |  | 0.935 | 0.458 - 1.828 | 0.748 |
| UBE2A | **1.531** | **1.008 - 2.374** | **0.013** |  | 0.926 | 0.550 - 1.446 | 0.6 |
| *NER* normalized expression ratio, *MDD* major depressive disorder | | | | | | |  |
| * Significant *p*-values set at 0.05 provided by REST software | | | | | |  |  |
