# Supplementary material for: Distinct Expression Pattern of Epigenetic Machinery Genes in Blood Leucocytes and Brain Cortex of Depressive Patients
Source: Mol Neurobiol. 2018 Oct 30;56(7):4697–707. doi: 10.1007/s12035-018-1406-0 (PMC6647377; doi:10.1007/s12035-018-1406-0)
Supplement: Supplementary file 4 — (DOCX 12 kb) [file 12035_2018_1406_MOESM4_ESM.docx]

| **Table S4** Normalized expression ratio (NER) of the candidate genes in the blood leucocytes of the MDD patients relative to the control subjects | | | |
| --- | --- | --- | --- |
| **Gene** | **NER** | **Std. Error** | ***p*-value*** |
| HDAC2 | **1.374** | **0.835 - 2.301** | **0.016** |
| HDAC4 | 0.961 | 0.472 - 1.959 | 0.849 |
| HDAC5 | 0.887 | 0.509 - 1.481 | 0.397 |
| HDAC6 | 0.961 | 0.495 - 1.881 | 0.834 |
| HDAC8 | 0.914 | 0.498 - 1.681 | 0.567 |
| DNMT1 | 1.267 | 0.767 - 2.125 | 0.102 |
| DNMT3A | 1.058 | 0.492 - 2.372 | 0.772 |
| DNMT3B | 0.727 | 0.354 - 1.471 | 0.085 |
| KAT2A | 0.856 | 0.304 - 2.881 | 0.579 |
| EHMT2 | 1.086 | 0.530 - 2.442 | 0.651 |
| UBE2A | 1.105 | 0.753 - 1.669 | 0.417 |
| *NER* normalized expression ratio, *MDD* major depressive disorder | | | |
| * Significant *p*-values set at 0.05 provided by REST software | | | |
